# Magnetic Heterodyne Target Proximal Distance Estimate Using Extended N-th-Pole Magnetic Dipole Model via Iterative Extended Kalman Filter

**DOI:** 10.3390/s26092792

**Published:** 2026-04-30

**Authors:** Xuyi Miao, Yipeng Li, Zumeng Jiang, Shaojie Ma, He Zhang, Peng Liu, Keren Dai

**Affiliations:** School of Mechanical Engineering, Nanjing University of Science and Technology, Nanjing 210094, China; xuyimiao@njust.edu.cn (X.M.); liyipeng@njust.edu.cn (Y.L.); jzm596@njust.edu.cn (Z.J.); shaojiem@njust.edu.cn (S.M.); njustca@njust.edu.cn (P.L.)

**Keywords:** magnetic dipole model, Kalman Filter, magnetic anomaly detection

## Abstract

Anti-collision detection technologies primarily rely on optical, radar, or laser sensors; however, their performance often deteriorates severely under adverse weather conditions (e.g., rain, snow, fog) or in scenarios involving visual occlusion. By contrast, magnetic anomaly detection leverages perturbations in the geomagnetic field induced by target objects (e.g., vehicles, metallic obstacles), exhibiting intrinsic all-weather operability and strong anti-interference capability. Nevertheless, conventional magnetic anomaly detection methods suffer from the limited applicability of the magnetic dipole model, which only affords coarse positioning accuracy and is predominantly suited for long-range targets. To address this limitation, this paper proposes an Extended N-th-Pole Magnetic Dipole (E-NMD) model that improves accuracy by analyzing the Lagrangian cosine term and rigorously constraining truncation errors under specific operational conditions. Experimental results demonstrate that, for steel with a relative permeability of 200, the model achieves a fitting variance of 99.87%. Furthermore, to overcome the inversion difficulties arising when the strength of short-range magnetic anomalies is comparable to sensor noise, an Adaptive Iterative Extended Kalman Filter (AI-EKF) is developed to enable robust noise suppression and precise distance estimation. Results indicate that E-NMD outperforms the traditional N-th-Pole Magnetic Dipole (NMD) model in proximal state estimation, achieving a 39.62% reduction in Root Mean Square Error (RMSE). Finally, in light of parameter uncertainty in magnetic anomaly targets under real-world conditions, a Dual-Mode Pairwise Iterative Extended Kalman Filter (DI-EKF) is introduced to jointly estimate parameters and system states, yielding an 89% reduction in RMSE compared to AI-EKF.

## 1. Introduction

The integration of magnetic anomaly detection [1,2,3] into anti-collision systems holds substantial promise. Conventional sensors, based on optics, radar [4,5], or lasers [6,7], are prone to significant performance degradation under adverse weather conditions (e.g., rain, snow, fog) or in environments with visual occlusion. In contrast, magnetic anomaly detection exploits perturbations of the geomagnetic field induced by target objects such as vehicles or metallic obstacles [8,9,10,11,12,13], providing robust all-weather and anti-interference capabilities. This technology effectively compensates for the blind zones of traditional sensors, particularly in low-speed and structurally complex urban environments (e.g., tunnels, underground car parks). This enables the accurate detection of stationary vehicles and metallic obstacles that would otherwise remain hidden, thus providing redundant sensory data to decision-making systems. Through multi-sensor fusion [14,15,16,17], magnetic anomaly detection can significantly enhance the reliability of the environmental perception of low-speed operations in harsh environments, such as collision avoidance during autonomous vehicle parking and collision detection when opening car doors after parking.

However, conventional magnetic anomaly detection techniques are predominantly designed for long-range scenarios [18,19,20,21], where the target is approximated as a point mass with a magnetic moment [22,23,24,25]. This modeling assumption significantly constrains their applicability to close-range detection tasks in unmanned systems. The key limitation arises from the use of the single magnetic dipole model, which assumes that the magnetic field strength decays proportionally to the cube of the distance when the observation point lies at more than 2.5 times the maximum dimension of the target. However, in close-range scenarios, the observation distance is often smaller than this threshold, rendering the dipole approximation invalid.

To address this limitation, Saber et al. developed a three-dimensional mathematical model of magnetic anomaly targets that incorporates the effects of higher-order poles, thereby extending applicability to short-range detection [18]. Nevertheless, their unmodeled derivation neglects critical factors such as higher-order truncation errors and measurement noise, which restrict the robustness and accuracy of the model in practical applications.

Another major challenge arises from the inherently weak strength of geomagnetic signals. In many application contexts, such as autonomous driving, magnetic anomaly detection suffers from low signal-to-noise ratios (SNRs), which substantially compromise detection accuracy. To mitigate this, Zhao et al. employed a sensor fusion approach that combined ultrasonic sensors and magnetometers, applying an Extended Kalman Filter (EKF) to reduce cumulative errors and improve the accuracy of position and orientation estimates [24]. Similarly, Liu et al. introduced a novel method for estimating the distance between dipoles and sensor arrays, demonstrating through simulations a 43% expansion of the successful positioning area [19]. Despite these advances, existing approaches remain constrained by the single-dipole assumption and are thus unsuitable for short-range detection in autonomous systems.

To overcome these technical bottlenecks, this paper makes the following contributions:

(1) Extended N-th-Pole Magnetic Dipole (E-NMD) Model: We establish a higher-order magnetic anomaly model by analyzing the truncation error. The proposed E-NMD extends the validity of conventional multipole models to close-range detection scenarios, thereby improving the inversion accuracy of the magnetic induction field.
(2) Adaptive Iterative Extended Kalman Filter (AI-EKF): To address the challenge of low SNR in close-range scenarios, we design an AI-EKF algorithm. This approach adaptively adjusts process and measurement noise covariances, enabling robust signal extraction and precise distance estimation. Compared with the traditional NMD model, the proposed method improves the Root Mean Square Error (RMSE) of distance estimation by 39.62%.
(3) Dual-Mode Pairwise Iterative Extended Kalman Filter (DI-EKF): Considering parameter uncertainties of magnetic anomaly targets under real-world conditions, we extend AI-EKF to a dual-mode structure. The DI-EKF simultaneously estimates both states and parameters, further improving stability and accuracy. Experimental validation shows that DI-EKF achieves an 89% improvement in RMSE over AI-EKF.

## 2. Methods and Modeling

### 2.1. Derivation of an Extended N-th-Pole Magnetic Dipole (E-NMD) Model

In autonomous driving, the accurate detection of proximal magnetic anomaly targets is of paramount importance for collision avoidance, particularly under harsh environmental conditions, where optical or vision-based sensors may fail. However, the application scope of the conventional single-dipole model is insufficient for such close-range scenarios. This necessitates the development of an enhanced mathematical formulation of magnetic induction strength that remains valid at short distances.

**Figure 1:**
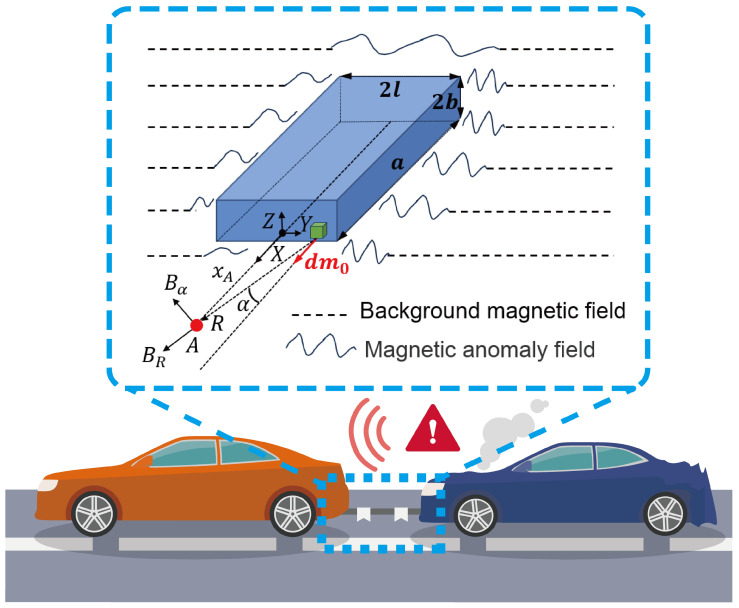
The conceptual framework of proximal magnetic anomaly detection for autonomous vehicle collision avoidance.

As illustrated in Figure 1, the coordinate system is established with the observation point A lying on a planar surface. The magnetic induction generated by a dipole at a single infinitesimal node established by Knoepfel [1] can be expressed as

(1) $$B_{R}=\frac{\mu_{0}dm_{0}}{2\pi R^{3}}\cos\alpha$$

(2) $$B_{\alpha }=\frac{\mu_{0}dm_{0}}{4\pi R^{3}}\sin\alpha$$

where:

$\mu_{0}$ denotes the magnetic permeability of free space;

$dm_{0}$ represents the differential magnetic moment of the node;

α is the angle between the magnetic moment $dm_{0}$ and the vector from the magnetic dipole to the observation point A;

*R* denotes the distance between the node and the observation point A;

$B_{R}$ denotes the component of the magnetic induction intensity in the vector;

$B_{\alpha }$ denotes the component of the magnetic induction intensity perpendicular to the vector.

Since the objective is to determine the distance of the observation point along the X-axis, the specific expression can be obtained by projecting the magnetic induction intensity in polar coordinates onto the X-axis by substituting Equations (1) and (2) into Equation (3):

(3) $$B_{x}=B_{R}\cos\alpha -B_{\alpha }\sin\alpha =\frac{\mu_{0}dm_{0}}{4\pi R^{3}}\left(2\cos^{2}\alpha -\sin^{2}\alpha \right)$$

By projecting the coordinates and considering that the primary motion occurs along the X-axis, the analysis of magnetic induction components along other axes is omitted here.

For a two-dimensional magnetic anomaly target, the total induction can be considered as the superposition of contributions from multiple dipoles. Integrating Equation (3) over the YZ-plane yields

(4) $$B_{x-fs}=\frac{\mu_{0}dm_{0}}{4\pi R^{3}}\int_{-l}^{l}\int_{-b}^{b}\frac{2x_{A}^{2}-\left(y_{A}^{2}+z_{A}^{2}\right)}{{\left(y_{A}^{2}+z_{A}^{2}+x_{A}^{2}\right)}^{\frac{5}{2}}}dydz$$

Further extending to three-dimensional targets, the total induction is obtained by integrating Equation (4) along the X-axis:

(5) $$B=\int_{-a}^{0}B_{x-fs}dx$$

(6) $$B=\frac{\mu_{0}mlb}{\pi }\left(\frac{1}{x_{A}{\left(b^{2}+x_{A}^{2}\right)}^{\frac{1}{2}}}-\frac{1}{\left(x_{A}+a\right){\left(b^{2}+{\left(x_{A}+a\right)}^{2}\right)}^{\frac{1}{2}}}\right)$$

where *m* denotes the magnetic moment per unit volume.

In practical scenarios, the observation distance along the X-axis is much smaller than the target dimension along that axis $\left(x_{A}+a\gg x_{A}\right)$. Under this condition, Equation (6) can be simplified as

(7) $$B=\frac{\mu_{0}mlb}{\pi }\frac{1}{x_{A}{\left(b^{2}+x_{A}^{2}\right)}^{\frac{1}{2}}}=\frac{\mu_{0}ml}{\pi }\frac{1}{x_{A}{\left(1+{\left(\frac{x_{A}}{b}\right)}^{2}\right)}^{\frac{1}{2}}}$$

However, Equation (7) remains overly complex for direct inversion. By performing a Taylor series expansion and retaining the first two terms while explicitly accounting for truncation error, we obtain the following approximation:

(8) $${\left(1+{\left(\frac{x_{A}}{b}\right)}^{2}\right)}^{\frac{-1}{2}}=1-\frac{1}{2}{\left(\frac{x_{A}}{b}\right)}^{2}+\frac{3}{8}{\left(\frac{x_{A}}{b}\right)}^{4}-\frac{5}{16}{\left(\frac{x_{A}}{b}\right)}^{6}+\ldots$$

(9) $$B=\frac{\mu_{0}ml}{\pi }\frac{1}{x_{A}{\left(1+{\left(\frac{x_{A}}{b}\right)}^{2}\right)}^{\frac{1}{2}}}=\frac{\mu_{0}ml}{\pi }\frac{1}{x_{A}}-\frac{x_{A}}{2b^{2}}$$

By parameterizing selected scalar terms in Equation (9), the magnetic induction of a 3D magnetic anomaly target can be expressed as

(10) $$B=\frac{p}{x_{A}}-WX_{A}+q$$

(11) $$p=\frac{\mu_{0}ml}{\pi }$$

(12) $$W=\frac{\mu_{0}ml}{2\pi b^{2}}$$

where *q* represents the background geomagnetic field.

To ensure robustness across a wide range of conditions, the final expression is formulated as a piecewise function:

(13) $$\left\{\begin{array}{c} B=B_{stat},x\geq x_{th}\\ B=\frac{p}{x}-Wx+q,x\leq x_{th} \end{array}\right.$$

This expression enables the derivation of specific formulations for different components ($B_{stat}$, $x_{th}$, *x*), as shown in Equations (14)–(16), which provide theoretical support for the subsequent design of DI-EKF.

(14) $$B_{stat}=\frac{p}{x_{th}}-Wx_{th}+q$$

(15) $$x_{th}=\frac{q-B_{stat}+\sqrt{{\left(q-B_{stat}\right)}^{2}+4WP}}{2W}$$

(16) $$x=\frac{q-B+\sqrt{{\left(q-B\right)}^{2}+4WP}}{2W}$$

The derivation of the E-NMD model for a rectangular prism in the near-field has been completed above. For targets with non-standard shapes, the E-NMD model can be obtained through the linear superposition of multiple rectangular prisms by segmenting the target, which is not further elaborated here.

### 2.2. AI-EKF for Distance Noise Estimation

**Figure 2:**
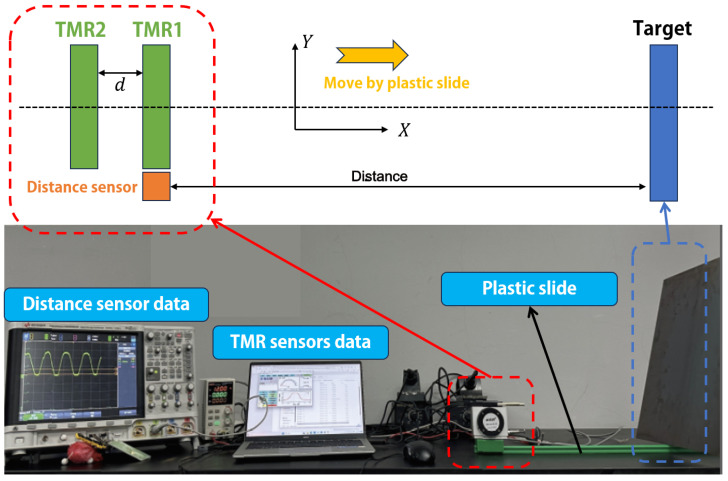
Configuration of the experimental proximal sensing system. The sensor array moves by green slide.

From the preceding derivation, the mathematical relationship between the distance of a single observation point and the magnetic induction strength of a proximal anomaly target has been established. Since Equation (13) contains multiple unknown parameters, the inversion of the E-NMD model requires the incorporation of additional observation constraints. As illustrated in Figure 2, two Tunneling Magnetoresistance (TMR) magnetometers (USB27053, Jiangsu Multi-Dimension Technology Co., Ltd. Measurement Range of −50 Oe to 50 Oe, RMS Noise 0.35 mOe. Suzhou, China) are deployed with spacing *d*, providing measurements $B_{1m}\left(t_{k}\right)$, $B_{2m}\left(t_{k}\right)$. The *X*-axial distance between the detection point and the target, $x(t_{k})$, is simultaneously obtained using a distance sensor (IP68 BRT38, Shenzhen BRITER Technology Co., Ltd. Shenzhen, China). The *d* between the TMR sensors is set as 50 mm. The target is a Q235/A3 steel plate with dimensions of 400 × 400 × 2 mm. The sensor array moves by the green plastic slide to get different magnetic field data with different distances. The dual-TMR configuration is employed to facilitate magnetic gradiometry, which effectively filters out ambient geomagnetic noise and enhances the spatial gradient sensitivity.

Because E-NMD is inherently nonlinear and the primary objective is the inversion of the target distance, an Iterative Extended Kalman Filter (IEKF) is employed. However, in practical experiments, the magnetic field magnitude of the anomaly target is often comparable to the noise level of the TMR sensors. Combined with the presence of background interference, this condition significantly reduces the detectability of signal features. To overcome this challenge, noise estimation must be integrated into the filtering process.

The Adaptive Iterative Extended Kalman Filter (AI-EKF) is therefore proposed, incorporating a feedback adjustment mechanism that adaptively modifies process and measurement noise covariances. The variable is shown in Table 1. This ensures the filter maintains higher fidelity to the true system states under noisy measurement conditions. The AI-EKF is designed as the equation of state $X\left(t_{k}\right)=\left[x\left(t_{k}\right)\right]$, and the measurement equation is shown as follows.

System dynamics:

(17) $$X\left(t_{k}\right)=X\left(t_{k-1}\right)+v\left(t_{k-1}\right),v∼\left(0,Q\right)$$

Measurement vector:

(18) $$Z=\left(\begin{array}{c} B_{1m}\left(t_{k}\right)\\ B_{2m}\left(t_{k}\right) \end{array}\right)$$

Measurement function:

(19) $$h\left(x_{k}\right)=\left\{\begin{array}{c} \frac{p}{x(t_{k})}-Wx\left(t_{k}\right)+q+n_{1}\left(t_{k}\right)\\ \frac{p}{x(t_{k})+d}-W\left(x\left(t_{k}\right)+d\right)+q+n_{2}\left(t_{k}\right) \end{array}\right.$$

where $v(t_{k})$ is the process noise; $n_{1}∼\left(0,R_{1}\right)$, $n_{2}∼\left(0,R_{2}\right)$ are the measurement noise; *d* is the distance between the TMR1 and TMR2.

The Jacobi matrix and update equations for the AI-EKF are shown below:

(20) $$h_{k}=\left(\begin{array}{c} \frac{p}{\hat{X_{k}}}-W\hat{X_{k}}+q+n_{1}\\ \frac{p}{\left(\hat{X_{k}}+d\right)}-W\left(\hat{X_{k}}+d\right)+q+n_{2} \end{array}\right)$$

(21) $$H_{k}=\left(\begin{array}{c} \frac{-p}{{\hat{X_{k}}}^{2}}-W\\ \frac{-p}{{\left(\hat{X_{k}}+d\right)}^{2}}-W \end{array}\right)$$

(22) $$C=H_{k}\hat{P_{k}}H_{k}^{T}+R$$

(23) $$K_{k}=\hat{P_{k}}H_{k}^{T}C^{-1}$$

(24) $${\hat{X}}_{k+1}={\hat{X}}_{k}+K_{k}\left[Z_{k}-h_{k}\right]$$

(25) $$P_{k+1}=\left(I-K_{k}H_{k}\right)P_{k}$$

The pseudo-code structure of the AI-EKF algorithm is shown in Algorithm 1.
**Algorithm 1** AI-EKF Algorithm  1:**Initialization**  2:**for** k=1:N **do**  3:      **Predict state and covariance** $\hat{X_{k}}$,$\hat{P_{k}}$  4:      Compute the Jacobians $H_{k}$  5:      Update $Z_{k}$,$h_{k}$  6:      Compute $K_{k}$  7:      Update $\hat{X_{k+1}}$,$\hat{P_{k+1}}$  8:      if k>windowsize  9:      **Compute** $C=\left[Z_{k}-h_{k}\right]{\left[Z_{k}-h_{k}\right]}^{T}/windowsize$10:      $\hat{R}=C-H_{k}\hat{P_{k}}H_{k}^{T}$11:      $\hat{R_{1}}=\alpha \hat{R_{1}}+\left(1-\alpha \right)\left|R\left(1,1\right)\right|$12:      $\hat{R_{2}}=\alpha \hat{R_{2}}+\left(1-\alpha \right)\left|R\left(2,2\right)\right|$13:      $\hat{Q}=\alpha \hat{Q}+\left(1-\alpha \right)\left(K_{k}CK_{k}^{T}\right)$14:      ${\hat{\theta }}_{k+1|k+1}={\hat{\theta }}_{k+1|k}+K_{k+1}^{\theta }\left[\Phi Z_{k+1}-\Phi h\left({\hat{X}}_{k+1|k}\right)\right]$15:      Adaptively adjust $R_{1},R_{2},Q$16:**end for**17:**End Loop**

### 2.3. Dual-Mode Pairwise Iterative Extended Kalman Filter (DI-EKF)

The preceding section demonstrated that the proposed AI-EKF substantially enhances estimation accuracy by adaptively adjusting noise covariances. However, its effectiveness diminishes under long-range conditions due to the inherent limitations of the E-NMD model—in particular, the presence of coefficients $B_{stat}$ and $x_{th}$, which introduce significant bias when the distance exceeds the threshold defined in Equation (13). Furthermore, the parameter values of the E-NMD model are highly target-dependent, varying across materials and approach directions. Consequently, robust estimation requires the simultaneous determination of both system states (e.g., distance, velocity, acceleration) and model parameters.

**Figure 3:**
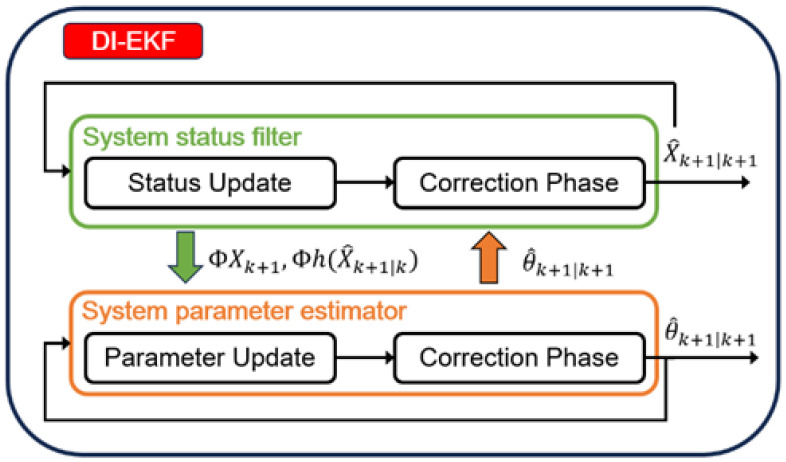
Schematic diagram of the DI-EKF algorithm flow.

To address these challenges, we design a Dual-Mode Pairwise Iterative Extended Kalman Filter (DI-EKF). This method introduces a coupled estimation framework, in which the state filter and parameter estimator operate jointly. The parameter updates are integrated into the iterative process, thereby ensuring that both system dynamics and structural parameters converge toward their true values. A schematic of the algorithm flow is presented in Figure 3. The variables are shown in Table 2.

The DI-EKF is designed as follows: the state variable *X* contains the distance x,v is the velocity of the measured object, a is the acceleration of the measured object, *F* is the computational matrix of the process state variables, $w_{k}$ is the process noise, and $Q_{k}$ is the covariance matrix of the process noise, the parameter θ, and the measurement equation *Z*.

(26) $$X=\left(\begin{array}{ccc} x & v & a \end{array}\right)$$

(27) $$\theta =\left(\begin{array}{ccc} p & W & q \end{array}\right)$$

(28) $$X_{k}=FX_{k-1}+w_{k}^{X},w_{k}^{X}∼\left(0,Q_{k}^{X}\right)$$

(29) $$\theta_{k}=F\theta_{k-1}+w_{k}^{\theta },w_{k}^{\theta }∼\left(0,Q_{k}^{\theta }\right)$$

(30) $$Z=\left(\begin{array}{c} B_{1}\\ B_{2} \end{array}\right)$$

(31) $$Z=h\left(X,\theta \right)+n_{k},n_{k}∼\left(0,R_{k}\right)$$

Since the measurement equations $B_{1},B_{2}$ are nonlinear, the Jacobi matrix with respect to the parameters is needed, which is as follows:

(32) $$H_{k}^{X}=\frac{\partial h_{k}}{\partial X}|{\hat{X}}_{k}^{-}$$

(33) $$H_{k}^{\theta }=\frac{\partial z}{\partial \theta }|{\hat{\theta }}_{k}^{-}$$

The flow of the algorithm of DI-EKF; its pseudo-code is as in Algorithm 2.
**Algorithm 2** DI-EKF Algorithm     1:**Initialization**     2:Initialize system states and structural parameters.     3:${\hat{X}}_{0|0}=E\left(X_{0}\right)$     4:$P_{0|0}^{X}=E\left[\left(X-{\hat{X}}_{0}\right){\left(X-{\hat{X}}_{0}\right)}^{T}\right]$     5:${\hat{\theta }}_{0|0}=E\left(\theta_{0}\right)$     6:$P_{0|0}^{\theta }=E\left[\left(\theta -{\hat{\theta }}_{0}\right){\left(\theta -{\hat{\theta }}_{0}\right)}^{T}\right]$     7:**for** k=1:N **do**     8:       **Predict state and error covariance**     9:       ${\hat{X}}_{k+1|k}={\hat{X}}_{k|k}+w_{k}^{X}$   10:       ${\hat{\theta }}_{k+1|k}={\hat{\theta }}_{k|k}+w_{k}^{\theta }$   11:       ${\hat{P}}_{k+1|k}^{X}=\Gamma_{1}{\hat{P}}_{k|k}^{X}\Gamma_{1}+Q_{k}^{X}$   12:       ${\hat{P}}_{k+1|k}^{\theta }=\Gamma_{\theta }{\hat{P}}_{k|k}^{\theta }\Gamma_{\theta }+Q_{k}^{\theta }$ 13:   14:       **Filter Gain Update**   15:       $K_{k+1}^{X}=P_{k+1|k}{\left(H_{k+1|k}^{X}\right)}^{T}\Phi^{T}{\left(\Phi \left(H_{k+1|k}^{X}P_{k+1|k}{\left(H_{k+1|k}^{X}\right)}^{T}+R_{k+1}^{Z}\right)\Phi^{T}\right)}^{-1}$ 16:   17:       $K_{k+1}^{\theta }=P_{k+1|k}^{\theta }{\left(H_{k+1|k}^{\theta }\right)}^{T}{\left[H_{k+1|k}^{\theta }P_{k+1|k}^{\theta }{\left(H_{k+1|k}^{\theta }\right)}^{T}+R_{k}^{\theta }\right]}^{-1}$ 18:   19:       **Filtered estimating equations**   20:       ${\hat{X}}_{k+1|k+1}={\hat{X}}_{k+1|k}+K_{k+1}^{X}\left[\Phi Z_{k+1}-\Phi h\left({\hat{X}}_{k+1|k}\right)\right]$ 21:   22:       ${\hat{\theta }}_{k+1|k+1}={\hat{\theta }}_{k+1|k}+K_{k+1}^{\theta }\left[\Phi Z_{k+1}-\Phi h\left({\hat{X}}_{k+1|k}\right)\right]$ 23:   24:       **Calculate the posteriori estimation error covariance matrix**   25:       $P_{k+1|k+1}^{X}=\left(I-K_{k+1}^{X}\Phi H_{k+1}^{X}P_{k+1|k}\right){\left(I-K_{k+1}^{X}\Phi H_{k+1}^{X}\right)}^{T}+K_{k+1}^{X}\Phi R_{K+1}^{Z}\Phi^{T}{\left(H_{k+1}^{X}\right)}^{T}$ 26:   27:       $P_{k+1|k+1}^{\theta }=P_{k+1|k}^{\theta }-P_{k+1|k}^{\theta }K_{k+1}^{\theta }C_{k+1|k}^{\theta }$ 28:   29:**end for**   30:**End Loop**

## 3. Results and Discussion

### 3.1. Validation of E-NMD

**Figure 4:**
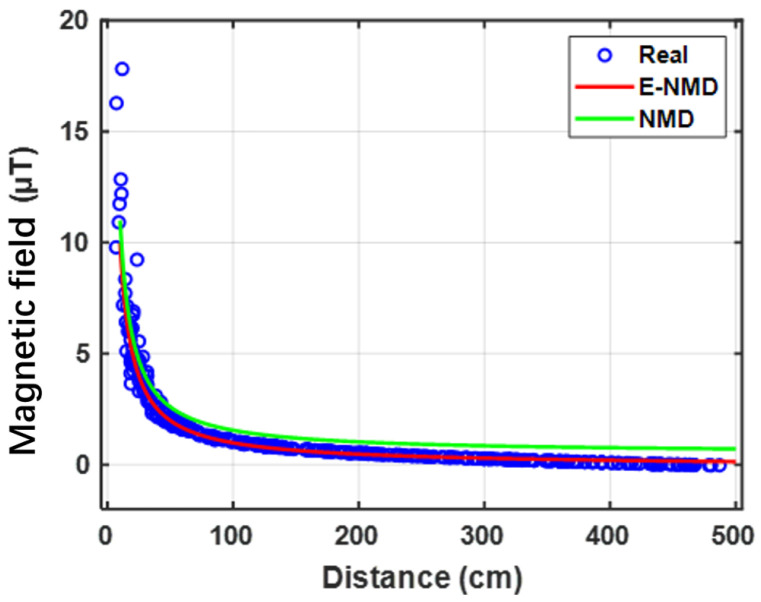
Performance evaluation of different magnetic field models.

By fitting magnetic induction data collected at different positions around the target with the proposed E-NMD model, the results (see Figure 4) demonstrate that the E-NMD achieves superior accuracy compared to the traditional NMD model, due to its refined treatment of truncation errors in Equation (8). To further validate the model, simulations were conducted in COMSOL 6.0 for different materials and approach directions. The results, summarized in Table 3 and Table 4, confirm that E-NMD consistently outperforms NMD. In particular, for steel (400 × 400 × 2 mm) with a relative permeability of 200, the fitting variance reached 0.9987, demonstrating the robustness and precision of the model.

### 3.2. Validation of AI-EKF

**Figure 5:**
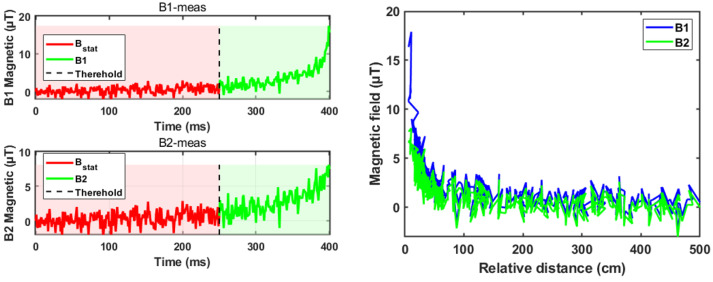
Spatial distribution of the magnetic field as the target approaches the sensor array.

**Figure 6:**
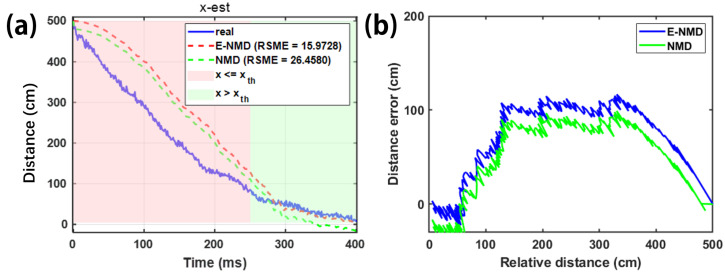
The model-switching mechanism and distance estimation of different models with time as the X-axis (**a**) and relative distance as the X-axis (**b**).

**Figure 7:**
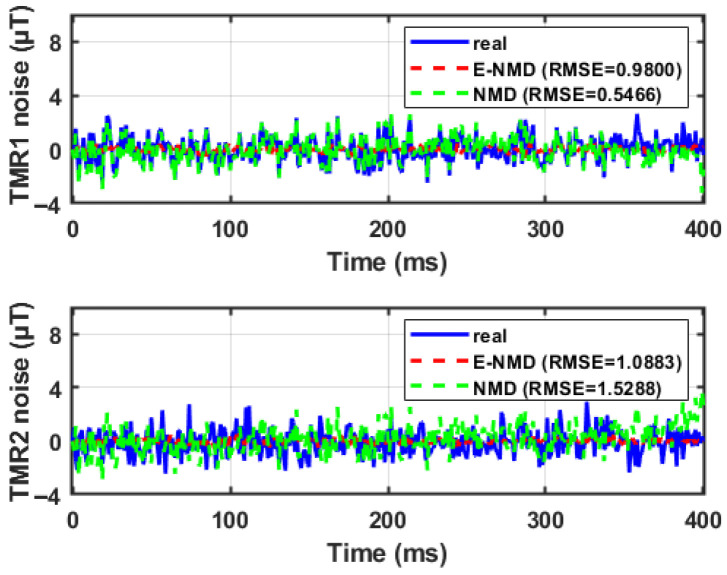
Sensor 1, 2 noise error estimation using different models.

As can be seen from Figure 5, in the actual measurement process, the test signals acquired by the sensors TMR1 and TMR2 are noisy, especially TMR2, whose noise amplitude can account for up to 50% of the total signal amplitude. This may be because TMR2 is further away from the magnetically dissimilar target during the actual measurement process, which results in a smaller amplitude of the signals, and at the same time because of the larger background noise since the signal-to-noise ratio is the lowest in the signals acquired by the TMR2. At the same time, due to the presence of the capital $B_{stat}$ in Equation (13), the magnetic induction strength signal can be considered almost unchanged until 250 ms, and the magnetic induction strength signal produces a significant change after 250 ms. As the distance approaches and the estimator runs iteratively, the error of the state estimation in Figure 6 decreases from 250 ms onwards (when $x\leq x_{th}$), which is because the measurement equations at this time are more in line with the mathematical model of the magnetic induction strength with $x\leq x_{th}$ in Equation (13), and because the estimation of the E-NMD model proposed in the paper is significantly better than the estimation of the NMD model. The RMSE improved by 39.62% after 250 ms, which proves that the accuracy of the E-NMD model proposed in the previous section is better than the traditional NMD model. The estimation for $n_{1},n_{2}$ in Figure 7 does not differ much, which is because the magnitude of the noise signal is determined by external factors such as the sensor itself, the background magnetic field, and so on, and has nothing to do with the chosen mathematical model. The design of the AI-EKF greatly reduces the influence of the noise signal, and the noise estimation effect of the AI-EKF has a certain degree of universality for the two models. The reason that makes the estimator perform well is the innovation of the feedback mechanism of sequence C is introduced to increase the adjustment of the noise covariance in real time under the condition of window_size, which greatly enhances the robustness of the estimator.

### 3.3. Validation of DI-EKF

**Figure 8:**
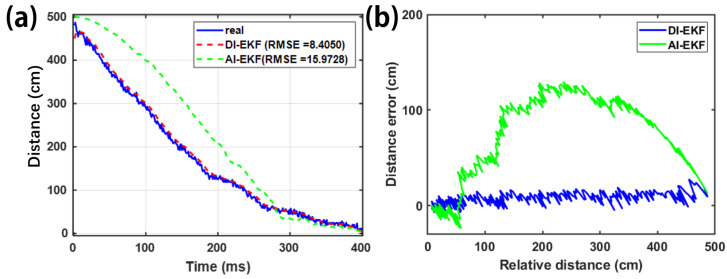
Distance estimation comparison of DI-EKF and AI-EKF with time as the X-axis (**a**) and relative distance as the X-axis (**b**).

From Figure 8, it can be seen that the real-time estimation and updating of parameter θ is added to DI-EKF to overcome the inaccuracy of AI-EKF estimation caused by $B_{stat}$ and $x_{th}$. This is because, for the data before threshold (i.e., $x\geq x_{th}$), the fluctuation range of $B_{stat}$ is very small, and the fitted true value of the parameter θ has an error between the calculated magnetic induction intensity and the real measured value under this condition, while the updating of the parameter θ is added to DI-EKF, along with the addition of the state transfer matrix *F*, both of which synergistically ensure the DI-EKF’s pre-threshold and post-threshold state estimation effect. In AI-EKF, the fixed fitting parameter θ has a large error in the estimation of the magnetic induction strength before the threshold, which leads to the poor estimation results of the AI-EKF estimator before the threshold, while the DI-EKF guarantees the accuracy of the state estimation before the threshold (i.e., $x\geq x_{th}$) through the addition of parameter θ and state transfer matrix F. At the same time, the state estimation results of the AI-EKF are not as accurate after the threshold (i.e., $x\leq x_{th}$), and the state estimation results of the AI-EKF are not as accurate after the threshold. Once B starts to change, it makes the proximal distance estimation go back to the AI-EKF state, which ensures the accuracy of the estimation after the threshold.

**Figure 9:**
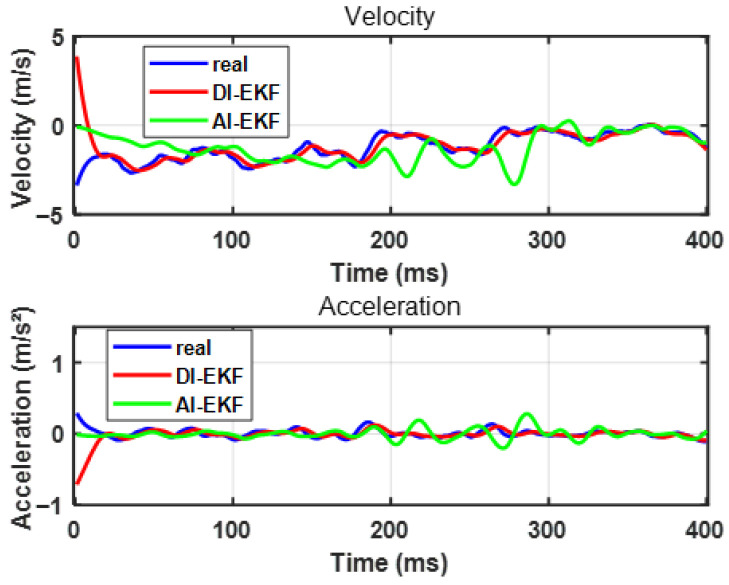
The velocity and acceleration estimation comparison of DI-EKF and AI-EKF.

**Figure 10:**
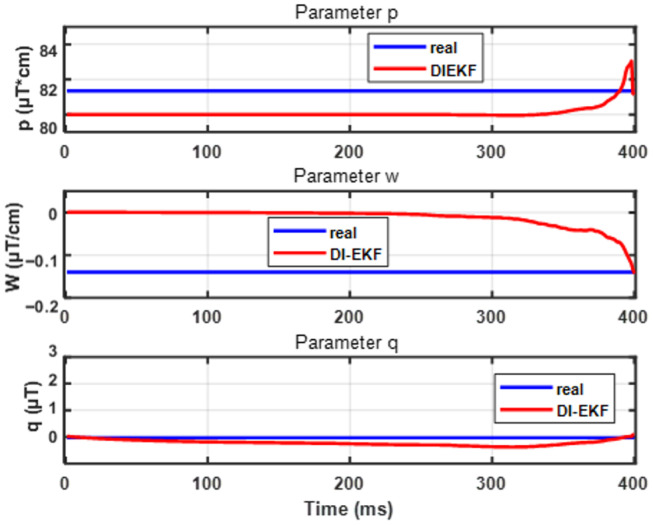
DI-EKF performance for estimation of parameters.

**Figure 11:**
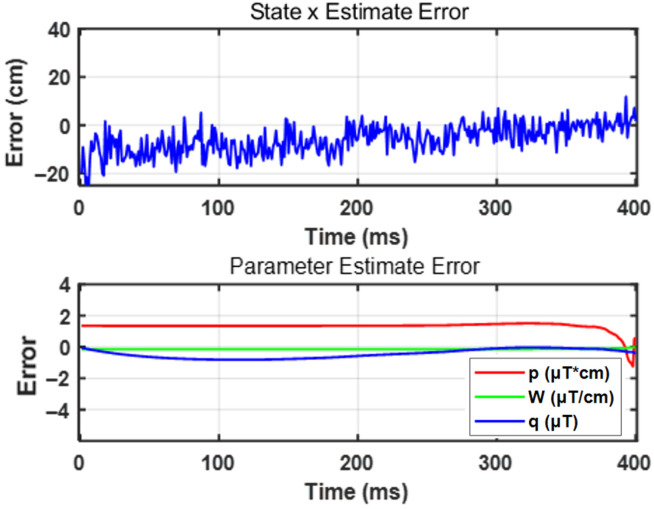
DI-EKF performance regarding estimation error for state and parameter.

Figure 9 shows that the estimation effect of DI-EKF for velocity and acceleration is better than that of AI-EKF, and AI-EKF does not add the real-time updated parameter θ to the prediction of displacement before 250 ms due to the existence of $B_{stat}$. At this time, the accuracy of the estimator is affected by $B_{stat}$, and the precision is affected by the signal-to-noise ratio of the measurement system, so the estimation of velocity and acceleration is more oscillating than that of AI-EKF are larger. As shown in Figure 10, for the parameter θ with the iterative operation of the estimator, the predicted update value slowly converges to the actual true value. Starting from the analysis of Equation (13), the parameter θ that affects B includes p,w,q, where p,w are the coefficients of the *x*-negative primary term and the primary term, and the two convergence states are affected by its influence. As can be seen in the figure, the magnitude of the value of p,w that changes with the iterative operation of the estimator before the threshold is very small. This is because, considering Equation (13), before the threshold, *x* changes greatly, while *B* is almost unchanged. At this time, if the coefficients related to *x* change, the accuracy of *B* will also be drastically changed with *x*, resulting in oscillations. At this time, the estimation effect will be very poor, and the magnitude of change of the two parameters of p,w before the threshold precisely illustrates that the DI-EKF estimator is not affected by the effect of the model before the threshold, but rather by updating q (and *x*-independent term) in real time to maintain the stability of the calculated intensity and the actual measured intensity. The stability of the state estimator before the threshold is guaranteed; for the post-threshold, the values of p,w change with the iterative running of the estimator by a large magnitude, but ultimately converge to the true value because of the computational model in Equation (13) after the threshold. The magnitude of the change in *x* is small, while the change in *B* is very large, and, at this time, it is necessary to maintain the stability of the calculated intensities with the actual measured intensities by adjusting the real values of the updated p,w. In summary, it is shown that DI-EKF makes the estimator robust and adaptive by adding the update of the parameter θ. Figure 11 then shows that, as the estimator runs iteratively, the errors of state estimation and parameter estimation slowly decrease, proving the convergence effect of the estimator.

## 4. Conclusions

This paper proposes an enhanced modeling and filtering framework for close-range magnetic anomaly detection in autonomous driving applications. The key contributions are threefold:

1. Extended N-th-Pole Magnetic Dipole Model (E-NMD): By analyzing the truncation error, the E-NMD significantly improves the accuracy of magnetic field inversion under short-range conditions. Simulation and experimental results confirm its robustness, with a fitting variance of 99.89% achieved for 0.4% carbon steel.
2. Adaptive Iterative Extended Kalman Filter (AI-EKF): To address the challenge of low signal-to-noise ratio, an AI-EKF algorithm was developed that adaptively adjusts noise covariances during estimation. The method effectively suppresses measurement noise and improves distance inversion accuracy, achieving a 39.62% reduction in RMSE compared with the conventional NMD model.
3. Dual-Mode Pairwise Iterative Extended Kalman Filter (DI-EKF): Considering the parameter uncertainty of magnetic anomaly targets in real-world conditions, DI-EKF was designed to jointly estimate both system states and model parameters. Experimental validation demonstrated an 89% RMSE improvement relative to AI-EKF, highlighting its strong adaptability and robustness.

Overall, the results confirm that reliable distance inversion can be achieved by exploiting short-range magnetic field perturbations, even under conditions where optical, radar, or laser sensors are unreliable (e.g., rain, snow, fog, or visual occlusion). The proposed E-NMD model, together with the AI-EKF and DI-EKF algorithms, provides a practical solution for enhancing the collision-avoidance capabilities of autonomous vehicles. In particular, the DI-EKF exhibits excellent adaptability, making it a promising approach for real-world deployment in adverse environments.

## Figures and Tables

**Table 1:** AI-EKF variable list.

Variable	Physical Deceleration	Unit
$x(t_{k})$	Relative distance	cm
$B_{1m}\left(t_{k}\right)$	TMR1 sensor data	μT
$B_{2m}\left(t_{k}\right)$	TMR2 sensor data	μT
$v(t_{k})$	Process noise	cm
*Q*	Covariance matrix	
$n_{1}\left(t_{k}\right)$	Measurement noise	μT
$n_{2}\left(t_{k}\right)$	Measurement noise	μT
$R_{1}$	Covariance matrix	
$R_{2}$	Covariance matrix	

**Table 2:** DI-EKF variable list.

Variable	Physical Deceleration	Unit
*x*	Relative distance	cm
*v*	Relative velocity	m/s
*a*	Relative acceleration	m/s^2^
*p*	Equivalent strength of the target magnetic source	μT·cm
*W*	The spatial gradient of the background magnetic field	μT/cm
*q*	Background geomagnetic field	μT
$w_{k}^{X}$	State *X* process noise	
$Q_{k}^{X}$	Covariance matrix	
$w_{k}^{\theta }$	State θ process noise	
$Q_{k}^{\theta }$	Covariance matrix	
$n_{k}$	Measurement noise	μT
$R_{k}$	Covariance matrix	

**Table 3:** Comparison of fitting results for different carbon steel materials (Y, Z, X represents steel with a relative permeability of 200; Y2, Z2, X2 represents steel with a relative permeability of 300).

Direction (Fitting Type)	*p*	*W*	*q*	$R^{2}$
Y (E-NMD)	0.218	0.00003	0.0046	0.9987
Y (NMD)	0.204		0.0027	0.9958
Z (E-NMD)	0.176	0.00005	0.0025	0.9687
Z (NMD)	0.202		0.0013	0.9572
X (E-NMD)	0.173	0.00013	0.0044	0.9681
X (NMD)	0.209		0.0009	0.9496
Y2 (E-NMD)	0.110	0.00008	0.0005	0.9897
Y2 (NMD)	0.114		0.0011	0.9889
Z2 (E-NMD)	0.180	0.00012	0.0029	0.9680
Z2 (NMD)	0.209		0.0013	0.9556
X2 (E-NMD)	0.038	0.00001	0.0006	0.9746
X2 (NMD)	0.045	0.00008	0.0002	0.9547

**Table 4:** Comparison of fitting results for different sizes of 200 permeability carbon steel materials (Y3, Z3, X3 represents size of 800 × 800 × 4 mm; Y4, Z4, X4 represents size of 200 × 200 × 1 mm).

Direction (Fitting Type)	*p*	*W*	*q*	$R^{2}$
Y3 (E-NMD)	0.206	0.00001	0.0024	0.9159
Y3 (NMD)	0.227		0.0001	0.8996
Z3 (E-NMD)	0.205	0.00001	0.0012	0.9695
Z3 (NMD)	0.218		0.0001	0.9638
X3 (E-NMD)	0.116	0.00004	0.0011	0.9468
X3 (NMD)	0.126		0.0004	0.9366
Y4 (E-NMD)	0.127	−0.00005	−0.0024	0.9847
Y4 (NMD)	0.113		−0.0009	0.9652
Z4 (E-NMD)	0.097	−0.00004	−0.0017	0.9850
Z4 (NMD)	0.087		−0.0007	0.9643
X4 (E-NMD)	0.061	−0.00002	−0.0012	0.9837
X14 (NMD)	0.055		−0.0004	0.9636

## Data Availability

No new data were created or analyzed in this study. Data sharing is not applicable to this article.
